# Quantum Binary Field Multiplication with Optimized Toffoli Depth and Extension to Quantum Inversion †

**DOI:** 10.3390/s23063156

**Published:** 2023-03-15

**Authors:** Kyungbae Jang, Wonwoong Kim, Sejin Lim, Yeajun Kang, Yujin Yang, Hwajeong Seo

**Affiliations:** Division of IT Convergence Engineering, Hansung University, Seoul 02876, Republic of Korea

**Keywords:** binary field, quantum multiplication, Toffoli depth, quantum inversion

## Abstract

The Shor’s algorithm can find solutions to the discrete logarithm problem on binary elliptic curves in polynomial time. A major challenge in implementing Shor’s algorithm is the overhead of representing and performing arithmetic on binary elliptic curves using quantum circuits. Multiplication of binary fields is one of the critical operations in the context of elliptic curve arithmetic, and it is especially costly in the quantum setting. Our goal in this paper is to optimize quantum multiplication in the binary field. In the past, efforts to optimize quantum multiplication have centred on reducing the Toffoli gate count or qubits required. However, despite the fact that circuit depth is an important metric for indicating the performance of a quantum circuit, previous studies have lacked sufficient consideration for reducing circuit depth. Our approach to optimizing quantum multiplication differs from previous work in that we aim at reducing the Toffoli depth and full depth. To optimize quantum multiplication, we adopt the Karatsuba multiplication method which is based on the divide-and-conquer approach. In summary, we present an optimized quantum multiplication that has a Toffoli depth of one. Additionally, the full depth of the quantum circuit is also reduced thanks to our Toffoli depth optimization strategy. To demonstrate the effectiveness of our proposed method, we evaluate its performance using various metrics such as the qubit count, quantum gates, and circuit depth, as well as the qubits-depth product. These metrics provide insight into the resource requirements and complexity of the method. Our work achieves the lowest Toffoli depth, full depth, and the best trade-off performance for quantum multiplication. Further, our multiplication is more effective when not used in stand-alone cases. We show this effectiveness by using our multiplication to the Itoh–Tsujii algorithm-based inversion of $F(x^{8}+x^{4}+x^{3}+x+1)$.

## 1. Introduction

Large-scale quantum computers in the near future are considered a major threat to the cryptography community. Quantum computers using quantum algorithms are expected to efficiently model and solve security problems that cryptographic algorithms are based on. Prominent quantum algorithms that can be used for cryptanalysis are the Grover search algorithm [1] and the Shor algorithm [2].

The Grover search algorithm is one of the leading quantum algorithms that reduces the classical search complexity of O(n) to the square root of *n* (i.e., $\sqrt{n}$). Grover’s search can decrease the security of symmetric key ciphers by a factor of the square root. In response, various symmetric key ciphers are being analysed under the Grover search algorithm [3,4,5,6]. It is worth noting that the quantum resources (elements that make up a quantum circuit such as quantum gates, qubits, and circuit depth) for applying the Grover’s search to AES [3,7,8,9] are being used as a standard for estimating post-quantum security strength by NIST [10].

In the cryptography community, it is considered that the threat of quantum computers is greater towards asymmetric key cryptography. Quantum computers equipped with the Shor algorithm are expected to solve the discrete logarithmic problems aand factorization of elliptic curve cryptography (ECC) and Rivest–Shamir–Adleman (RSA). Thus, it can be said that the Shor algorithm is the most powerful attack that can break the security of asymmetric key cryptography. In such circumstances, for a robust security system [11,12], analysis of potential quantum computer attacks on asymmetric key cryptography should be considered.

In [13], Häner et al. studied the quantum cryptanalysis of RSA under the Shor algorithm and presented a quantum circuit requiring 2n+2 qubits when an *n*-bit key is used. In another study, Gidney showed that applying the Shor algorithm [14] to RSA with an *n*-bit key requires 2n+1 qubits.

For quantum cryptanalysis of ECC, Roetteler et al. in Asiacrypt’17 [15] estimated the quantum resources required to solve discrete logarithm problems on elliptic curves. As a result, it was shown that ECC can be solved with fewer quantum resources than RSA. Later, Häner et al. improved the work of [15] in PQCrypto’20 [16], reducing the qubit count and circuit depth. Both studies [15,16] mainly optimized scalar multiplication for elliptic curves to reduce the cost of quantum attacks, and both targeted prime curves.

In CHES’20 [17], Banegas et al. presented a quantum analysis of binary curves and found that their method required fewer qubits and had a lower circuit depth than methods for prime curves. In their study, Banegas et al. utilized the technique developed by Van Hoof [18] to implement quantum multiplication in binary fields. Van Hoof’s quantum multiplication method, which is based on the Karatsuba algorithm, has a space-efficient implementation that reduces the qubit count and Toffoli gate count.

Optimizing binary field multiplication on a quantum computer is a crucial step towards achieving high-performance quantum cryptanalysis, as demonstrated in previous research. Most recent studies on quantum multiplication concentrate on reducing the use of qubits or Toffoli gates, but consider the circuit depth less [18,19,20,21,22]. Previously, quantum computers had a restricted qubit count, but contemporary quantum computers have grown significantly in size and capability. Additionally, it is clear that upcoming quantum computers will not be small, as indicated by IBM’s quantum computer development roadmap (https://research.ibm.com/blog/ibm-quantum-roadmap, accessed on 1 January 2023).

Toffoli depth is a major metric in quantum computing for correcting errors, and full depth determines the operating time of the circuit [23]. The full depth is a crucial factor in the calculation of the cost of quantum attacks according to the National Institute of Standards and Technology’s (NIST) post-quantum security requirements (https://csrc.nist.gov/CSRC/media/Projects/Post-Quantum-Cryptography/documents/call-for-proposals-final-dec-2016.pdf, accessed on 1 January 2023.) [10]. The cost of quantum attacks is determined by the product of the gate count and the full depth, with the number of qubits not being taken into account.

The aim of this paper is to optimize the multiplication of binary fields on a quantum computer (particularly in terms of Toffoli depth and full depth). We propose a quantum binary field multiplication that is optimized with a minimal Toffoli depth and has the lowest full depth. However, the proposed quantum multiplication requires the use of additional qubits. Therefore, to run our multiplication on an actual quantum computer, a quantum computer that can use many qubits is required (i.e., a large-scale quantum computer is needed). However, if we count the physical number of qubits rather than the number of logical qubits that do not cause errors, the depth also affects the number of qubits required for error correction.

In this trade-off, we evaluate our work using various metrics including the qubit count (*M*), quantum gates, Toffoli depth (TD), and full depth (FD). We seek to minimize these measures while still achieving good performance in quantum multiplication. As a result, our method provides the best performance in terms of the trade-off metrics of the product of Toffoli depth and qubit count (i.e., TD·M), and the product of full depth and qubit count (i.e., FD·M).

Most quantum-related research involves simulating quantum circuits on classical computers using quantum simulation tools, such as Qiskit [24], Q# [25], and ProjectQ [26]. These simulations can be performed at the logical level, so they do not take into account errors that may occur in actual quantum computation. Although it can be simulated on the physical level, the result of this paper focuses on the logical level. We utilize the quantum simulation tool ProjectQ to simulate our proposed quantum circuits, and to analyse the detailed quantum resources.

### 1.1. Our Contribution

This paper makes the following contributions:

1. Optimization of quantum binary field multiplication with the Karatsuba algorithm (Section 3.1 and Section 3.2). We have developed an optimized method for performing quantum binary field multiplication using a quantum circuit with a Toffoli depth of one. The Toffoli gates in this circuit operate in parallel, allowing all products to be generated simultaneously. The full depth of quantum multiplication is primarily determined by the depth of Toffoli gates. Our proposed method for quantum multiplication has a Toffoli depth of one, which naturally reduces the full depth of the multiplication.
2. Efficient techniques for implementing quantum circuits. We present efficient implementation techniques, including methods for offsetting the overhead of increasing qubits in quantum multiplication (Section 3.3), using quantum multiplication with a T-depth of one (Section 3.4), and performing optimal modular reduction (Section 3.5).
3. Efficient implementation of quantum inversion built on the Itoh–Tsujii algorithm. By using the proposed quantum multiplication and efficient techniques, we implement quantum inversion built on the Itoh–Tsujii algorithm [27]. This demonstrates the effectiveness of our multiplication when used in non-stand-alone cases.
4. Optimization of primitives for quantum cryptanalysis of elliptic curve cryptography. Our work can be used to optimize the implementation of quantum arithmetic for cryptanalysis of elliptic curves over binary fields. Quantum binary field multiplication is a key component of the Shor algorithm to solve discrete logarithm problems on binary elliptic curves. Our work demonstrates the optimal balance between Toffoli depth and the number of qubits for this application.

### 1.2. Extended Version of WISA’22

The work presented in WISA’22 is revisited in this paper [28]. Efficient quantum multiplication of binary fields was presented in [28]. In this paper, the proposed quantum multiplication is analysed for larger (various) field sizes.

Further, this time we show the effectiveness of the proposed multiplication when not used in stand-alone cases. We present an efficient quantum circuit for inversion using our work in implementing the inner multiplications of Itoh–Tsujii-based inversion.

The source code (https://github.com/starj1023/Binary_mul, accessed on 1 February 2023) for our work is open to the public.

## 2. Background

This section lays out the groundwork for understanding our work. The multiplication of binary fields, the Karatsuba algorithm adopted in our implementation, quantum computing, and quantum gates for implementing quantum circuits are covered.

### 2.1. Multiplication of Binary Fields

Multiplication of $F_{2^{n}}$, with *n*-bit polynomials, performs modular reduction using an irreducible polynomial *N*. The multiplication of *f* and *g* in $F_{2^{n}}$ is described as follows.

(1) $$\begin{array}{c} h=f\cdot g\;\mathrm{mod}\;N \end{array}$$

The generated product of *f* and *g* is subjected to modular reduction over *n* bits in length, resulting in *h*, the product of *f* and *g*, becoming an element of $F_{2^{n}}$.

### 2.2. Karatsuba Algorithm

The Karatsuba algorithm [29] is well-known for its ability to simplify the process of multiplication through the use of addition. Using the Karatsuba algorithm, the two input polynomials *f* and *g* of *n* size (resulting in h=f·g) are divided into two halves of size s=n/2 as follows:

(2) $$\begin{array}{c} f=f_{1}x^{s}+f_{0}\\ g=g_{1}x^{s}+g_{0} \end{array}$$

After dividing the polynomials (*f* and *g*) as described, the Karatsuba algorithm proceeds with the multiplication as follows:

(3) $$f_{0}\cdot g_{0}+\left\{\left(f_{0}+f_{1}\right)\cdot \left(g_{0}+g_{1}\right)+f_{0}\cdot g_{0}+f_{1}\cdot g_{1}\right\}x^{s}+f_{1}\cdot g_{1}x^{2s}$$

By utilizing the Karatsuba algorithm, the complexity of multiplying polynomials is reduced from $O(n^{2})$ to $O(n^{\log_{2}3})$ through the use of addition operations.

### 2.3. Quantum Computing

Quantum computers utilize the properties of superposition and entanglement of qubits (quantum bits) to enable computing in a different dimension than classical computers. Quantum algorithms can efficiently model and solve problems that are difficult to solve on classical computers. However, quantum computers can also compromise security in symmetric key cryptography through Grover’s algorithm [1], and in public key cryptography through Shor’s algorithm [2]. To run quantum algorithms on quantum computers, classical operations need to be efficiently translated into the quantum domain. As a result, research is being conducted to optimize arithmetic operations used in cryptography for quantum computing [15,16,17,18,19,20,30].

### 2.4. Reversible Quantum Gates

**Figure 1:**
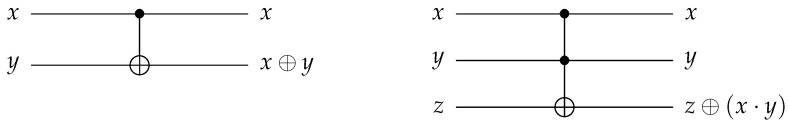
Quantum gates: CNOT (**left**) and Toffoli (**right**) gates.

Reversible quantum gates can compute a unique input from a given output by reversing the previous quantum gates. By performing the reverse operation on the output of the quantum gates in Figure 1, the original input can be recovered by reversing the previous quantum gates. This section briefly explains how the CNOT and Toffoli gates can be used to perform binary field multiplication, which involves XOR and AND operations.

The left side of Figure 1 shows a quantum CNOT gate, which can take the place of the classical XOR operation. In the CNOT gate, the value of the result qubit is controlled by the state of the control qubit (CNOT(x,y)=(x,x⊕y)). The right side of Figure 1 shows a quantum Toffoli gate, which can take the place of the classical AND operation. In the Toffoli gate, the value of the result qubit is controlled by the states of two control qubits (Toffoli(x,y,z)=(x,y,z⊕(x·y))).

**Figure 2:**
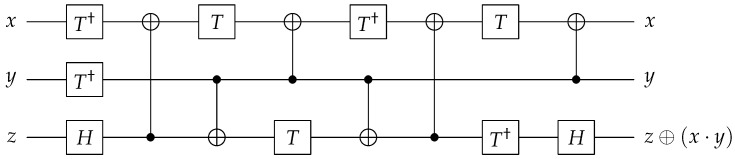
Decomposition of the Toffoli gate.

The Toffoli gate has a high implementation cost due to the combination of quantum gates required [31]. We use one of the decomposition methods in [31], 8 Clifford gates + 7 *T* gates, resulting in a full depth of eight and a *T*-depth of four for the Toffoli gate. Figure 2 shows the decomposition of the Toffoli gate. Since this decomposition method has been frequently adopted in previous works, we also employ it to facilitate a comparison of performances.

## 3. Quantum Binary Field Multiplication

In quantum multiplication, a significant portion of the cost is incurred by using Toffoli gates to compute products (i.e., AND operations). The Karatsuba algorithm reduces the number of multiplication operations and is therefore a highly efficient technique in quantum computers. That is, by reducing the number of AND operations (classical), we can optimize the Toffoli-related metrics (quantum).

We propose a special form of quantum Karatsuba multiplication that reduces the Toffoli gate count, Toffoli depth, and full depth, leading to more efficient quantum computation. By using this technique, we implement a quantum binary field multiplication that has a Toffoli depth of one and can simultaneously perform all multiplication operations. Our proposed quantum multiplication technique reduces the full depth of the quantum circuit by reducing the Toffoli depth in multiplication, which plays a key role in determining the full depth.

### 3.1. Parallel Quantum Multiplication Using the Karatsuba Algorithm

**Figure 3:**
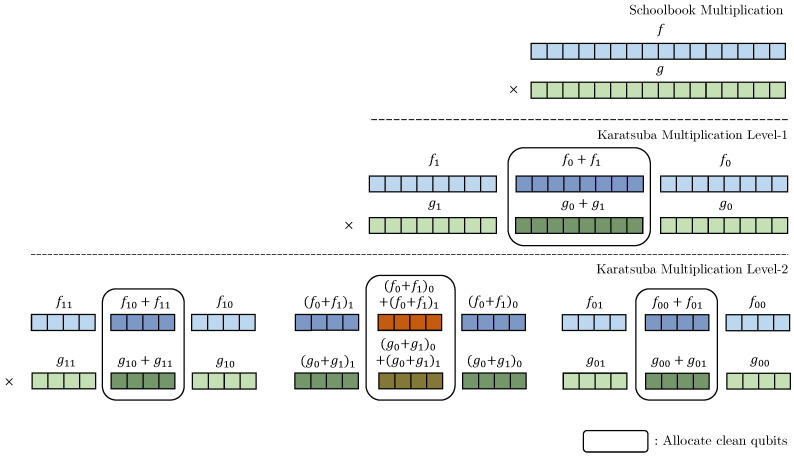
Overview of the proposed method.

Denote the product of two polynomials *f* and *g* of size *n* as *h* (i.e., h=f·g). As shown in Figure 3, using Schoolbook multiplication (which is a general method) to compute f·g requires $n^{2}$ Toffoli gates.

Our proposed method involves applying the Karatsuba algorithm once to reduce the size of the multiplication, which we refer to as Level-1. In Level-1, the multiplication is divided into three parts: $f_{0}\cdot g_{0}$, $f_{1}\cdot g_{1}$, and $\left(f_{0}+f_{1}\right)\cdot \left(g_{0}+g_{1}\right)$. The size of each multiplication is reduced to (n/2) and a total of $3\cdot {\left(n/2\right)}^{2}$ Toffoli gates are needed. This result reduces the number of Toffoli gates from $n^{2}$ to $3\cdot {\left(n/2\right)}^{2}$, but the three multiplications may not be performed simultaneously, meaning it may not be fully parallel. In the Level-1 layer shown in Figure 3, the multiplications $f_{0}\cdot g_{0}$ and $f_{1}\cdot g_{1}$ (the lower and upper parts of the multiplication, respectively) are performed simultaneously, but the multiplication $\left(f_{0}+f_{1}\right)\cdot \left(g_{0}+g_{1}\right)$ (the middle part of the multiplication) is carried out sequentially after the previous multiplications are completed. The operands of the multiplication in the middle part ($f_{0}+f_{1}$, $g_{0}+g_{1}$) can be overwritten in $f_{0}$, $g_{0}$ or $f_{1}$, $g_{1}$ (i.e., $f_{0}=f_{0}+f_{1}$, $g_{0}=g_{0}+g_{1}$) just as described in [18,20,21], but this can only occur after the multiplications in the upper and lower parts are completed (i.e., sequential).

In contrast to the lower and upper parts of the multiplication, the middle part requires a different approach. To begin the multiplications, we first allocate clean qubits (as shown in the rectangle in the Level-1 layer of Figure 3). Then we independently prepare the operands for the middle part of the multiplication, $\left(f_{0}+f_{1}\right)$ and $\left(g_{0}+g_{1}\right)$ to the clean qubits using CNOT gates. In order to prepare the middle part of the multiplication, *n* clean qubits and 2n CNOT gates are needed. Specifically, 2/n clean qubits are used for $f_{0}+f_{1}$ and 2/n clean qubits are used for $g_{0}+g_{1}$. Then, we can perform the low, middle, and high multiplications independently and simultaneously. This approach allows for a quantum multiplication method with reduced Toffoli depth in the Level-1 layer. If all products $f_{0}\cdot g_{0}$, $f_{1}\cdot g_{1}$, and $\left(f_{0}+f_{1}\right)\cdot \left(g_{0}+g_{1}\right)$ are generated, we can use CNOT gates to complete the Karatsuba multiplication by performing the remaining addition operations. However, we use the Karatsuba algorithm in a recursive manner to optimize the Toffoli and full depths (will be described in Section 3.2).

The quantum resources required for multiplication at each Karatsuba level are presented in Table 1. Actually, the Toffoli gate is typically decomposed (implemented) using a mixture of various quantum gates. In this work, we use a frequently adopted method [31] to decompose the Toffoli gate into 8 Clifford gates + 7 *T* gates, resulting in a full depth of eight and a *T*-depth of four. However, for a high-level comparison, in Table 1, only the full depth of the Toffoli gate is assumed to be eight without decomposing it. In the following resource estimates, we fully decompose the Toffoli gates.

Modular reduction is not included in the estimation presented in Table 1, and will be further discussed in Section 3.5. This is because the complexity of modular reduction may vary slightly depending on the specific irreducible polynomial of the field, but it does not have a significant impact on the cost.

### 3.2. Optimizing Toffoli Depth with the Recursive Karatsuba Algorithm

In Level-2, the Karatsuba algorithm is applied individually to each of the three multiplications that were divided into smaller parts in the previous level (i.e., Level-1). There is a dependency on the middle part of each of the multiplications (in Level-2), similar to the dependency on the middle multiplication in Level-1. We allocate new 3× clean qubits and use CNOT gates to prepare the middle parts again (shown as rectangles in the Level-2 layer of Figure 3) To prepare these middle parts, we need 3·(n/2) clean qubits and 3n CNOT gates. Through this process, the nine multiplications become totally independent from one another and can be performed simultaneously. In Level-2, the Toffoli gate count and the Toffoli depth that were reduced in Level-1 are further reduced to $3^{2}\cdot {\left(n/2^{2}\right)}^{2}$ and $\left(3n/2^{2}-2\right)$, respectively.

By using this method, the Karatsuba algorithm is applied recursively until the multiplications are reduced to size one (i.e., 1×1 multiplications), which allows all the dependencies between multiplications to be eliminated. As a result, we can perform quantum multiplication with a Toffoli depth of one by generating all the products in parallel. The quantum multiplication circuit that is proposed ultimately achieves the best performance with a Toffoli depth of one, even for full depth, resulting in high performance.

The required Karatsuba Level for multiplication with a Toffoli depth of one differs based on the field size $2^{n}$. This can be calculated as Level-$\log_{2}n$ for a field size of $2^{n}$. For example, the required Karatsuba Level is two for a field size of n=4 and three for a field size of n=8. In Table 2, we compare the quantum resources needed for multiplication with a Toffoli depth of one by field size. As the Karatsuba Level increases, there is a trade-off among the number of Toffoli gates, depth, and number of qubits (refer to Table 1). From Table 2, it can be seen that when the field size doubles, the qubit count triples. In contrast, it is observed that the full depth does not increase much because the Toffoli depth is always optimized at one.

The proposed quantum multiplication provides the best performance at the highest Karatsuba Level, but the implementation designer can adjust the Karatsuba Level based on this trade-off as desired.

### 3.3. Reusing Qubits through Reverse Operation

Our quantum multiplication method requires the allocation of new qubits for the middle parts each time the Karatsuba algorithm is applied, which incurs overhead. However, these qubits can be reset using the inverse operation of the CNOT gates previously applied to the middle parts.

The qubits are initialized after the Toffoli gates that generate the products have completed their operation. If all products are generated at the same time in the lowest layer, we reset (clean) the ancilla qubits from the lower layer to the upper layer by applying the reverse of the operations used to prepare the middle parts. In other words, to initialize the ancilla qubits, one simply needs to perform all the CNOT gates applied to these qubits in reverse. This initialization process causes the qubits allocated for the middle parts to be reset to the zero state.

This qubit cleaning process, called initialization, can be utilized when the multiplication is part of a larger computation (i.e., not a stand-alone multiplication). This means that in later multiplications, the initialized qubits from the previous multiplication can be reused instead of allocating new qubits for the middle parts. The initialized qubits can also be utilized for other operations that require clean qubits, not just multiplication. The qubit initialization method is particularly useful in cryptography because multiplication is a fundamental operation. We also found that the reverse operation used for initialization does not add to the circuit depth, as it can be carried out while the multiplication is taking place (e.g., during the process of combining products or performing modular reduction). This technique allows us to effectively mitigate the overhead of qubits in our quantum multiplication. In our multiplication, these ancilla qubits make up most of the total number of qubits. However, thanks to the initialization technique that enables reusability, we can significantly reduce the number of qubits by not allocating this many ancilla qubits for the following multiplications. By reusing qubits in subsequent multiplications, we only need 17, 43, 113, 2439, 4713, 6789, 10,839, and 32,313 qubits for n= 4, 8, 16, 127, 163, 233, 283, and 571, respectively, (compared to 27, 81, 243, 6555, 13,161, 18,969, 30,819, and 93,513 qubits without reuse). In Section 4, quantum inversion using this cleaning technique will be described.

### 3.4. T-Depth One Quantum Multiplication

**Figure 4:**
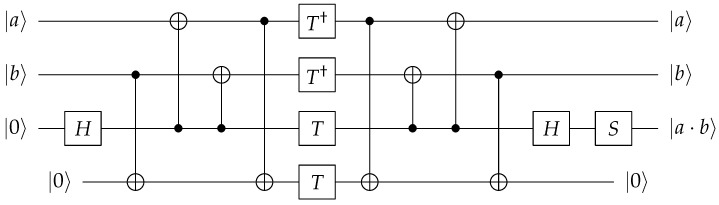
Quantum AND gate.

For the purpose of optimizing *T*-depth, we employ the quantum AND gate with a *T*-depth one from [7] instead of the Toffoli gate. In Figure 4, the AND gate uses a single ancilla qubit and has a *T*-depth one. After the AND gate is performed, the single ancilla qubit is initialized to zero and can be reused in subsequent AND gates. However, this reuse imposes a sequential operation of AND gates. We assign a new ancilla qubit for each AND gate, allowing them to operate in parallel. As a result, a quantum multiplication with *T*-depth of one is successfully implemented, with all AND gates operating in parallel.

**Figure 5:**
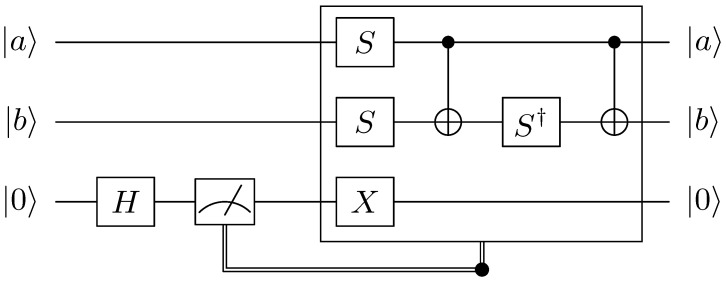
Quantum AND dagger gate.

AND gates require additional qubits, but these qubits are initialized at the end. The ancilla qubits that are used in AND gates can be reused in future multiplications or other operations that require clean qubits. This technique is similar to the method described in Section 3.3. The AND dagger gate (i.e., AND reverse gate) in Figure 5 is designed based on measurements and the *T* gate is not used. The quantum resources required for quantum multiplication of *T*-depth one using the AND gate are shown in Table 3.

### 3.5. Quantum Modular Reduction

Quantum multiplication can be customized to fit the modular reduction based on the irreducible polynomial of the field (actually, liner layer optimization). In this Section, we explore the customization of modular reduction for $F_{2^{8}}/\left(x^{8}+x^{4}+x^{3}+x+1\right)$ (field of S-box in AES) and the quantum resources required.

In a previous study [20], the authors eliminated the step of combining products and instead calculated the results using linear combinations of products following modular reduction. This means that the steps of combining and modular reduction are merged into a single step, and the coefficients are calculated. For the field $F_{2^{8}}/\left(x^{8}+x^{4}+x^{3}+x+1\right)$, merging the steps of combining and modular reduction into a single step and calculating the linear combinations of products requires 70 CNOT gates and a full depth of 27. However, if the combining step and modular reduction are kept separate and the coefficients are calculated separately, 85 CNOT gates are used and the full depth is 17 for $F_{2^{8}}/\left(x^{8}+x^{4}+x^{3}+x+1\right)$.

In our implementation, we choose to separate the combining step and modular reduction in order to prioritize reducing the depth over using more CNOT gates. This approach is more general and allows for greater flexibility in optimization. Efficient CNOT operations on linear combinations of combining are performed first (62 CNOT gates) and then efficient CNOT operations on linear combinations of modular reduction are completed (23 CNOT gates).

After completing the combining step in our quantum Karatsuba multiplication, we obtain 2n−1 products of $c_{0},c_{1},\ldots ,c_{2n-2}$. Furthermore, we customize the quantum implementation of modular reduction under the irreducible polynomial $x^{8}+x^{4}+x^{3}+x+1)$. The coefficients after performing modular reduction of $F_{2^{8}}/\left(x^{8}+x^{4}+x^{3}+x+1\right)$ are shown in Table 4. It is efficient to generate the elements with the same colours in Table 4 only once, and pass them (using only one CNOT gate) on as coefficients when preparing $x^{n}$. For example, to prepare the red-coloured $c_{8}=c_{8}+c_{9}+c_{14}$ in qubit $c_{8}$, we perform CNOT($c_{9},c_{8}$) and CNOT($c_{14},c_{8}$). Then, we can use qubit $c_{8}$ as a coefficient for $x^{4}$ and $x^{1}$ by performing CNOT($c_{8},c_{4}$) and CNOT($c_{8},c_{1}$). The naïve implementation needs 30 CNOT gates, while the customized version only requires 23 and has a reduced depth. The quantum resources needed for the multiplication in $F_{2^{8}}/\left(x^{8}+x^{4}+x^{3}+x+1\right)$, including the modular reduction are reported in Table 5.

Actually, modular reduction corresponds to linear operations. Furthermore, independent of this work, there are several papers dedicated to optimizing (heuristic-based) linear operations. These optimization techniques can be applied to modular reduction. However, to the best of our knowledge, there is no work to optimize large-size linear layers. Current linear operation optimization techniques cannot be applied to modular reduction when the field size is large.

## 4. Efficient Implementation of Quantum Binary Field Inversion

This section shows how effective the proposed quantum multiplication can be in implementing quantum inversion. As explained in Section 3.3, the ancilla qubits used within our quantum multiplication can be recycled, which is effective when used to implement quantum inversion. The proposed quantum inversion quantum circuit is implemented built on the Itoh–Tsujii algorithm [27]. So, the inverse of element *a* in $F_{2^{8}}/\left(x^{8}+x^{4}+x^{3}+x+1\right)$ is computed as: $a^{-1}=a^{254}={\left(\left(a\cdot a^{2}\right)\cdot {\left(a\cdot a^{2}\right)}^{4}\cdot {\left(a\cdot a^{2}\right)}^{16}\cdot a^{64}\right)}^{2}$. Multiple multiplications are required here, so the ancilla qubits used within the first multiplication are recycled until the last multiplication. In other words, the overhead of requiring many ancilla qubits does not apply except for the first multiplication. Thanks to this, the overhead for ancilla qubits is reduced while maintaining a low Toffoli depth and full depth.

For Squaring, modular reduction can be represented as a linear matrix and then implemented using LUP decomposition. Therefore, Squaring is implemented in place using only CNOT gates as described in Algorithm 1 ($x_{7}$ represents $x^{7}$). Squaring is not a major focus for optimizing quantum inversion due to the very few quantum resources required.
**Algorithm 1** Quantum circuit for Squaring of $F_{2^{8}}/\left(x^{8}+x^{4}+x^{3}+x+1\right)$.**Input:** $a(a_{7},a_{6},a_{5},a_{4},a_{3},a_{2},a_{1},a_{0})$**Output:** $a^{2}$1:CNOT($a_{4},a_{0}$), CNOT($a_{6},a_{0}$), CNOT($a_{5},a_{1}$)2:CNOT($a_{4},a_{2}$), CNOT($a_{7},a_{2}$), CNOT($a_{5},a_{3}$)3:CNOT($a_{6},a_{4}$), CNOT($a_{7},a_{4}$), CNOT($a_{6},a_{5}$)4:CNOT($a_{6},a_{7}$), CNOT($a_{4},a_{6}$), CNOT($a_{5},a_{6}$)5:**return** $a(a_{7},a_{3},a_{5},a_{2},a_{6},a_{1},a_{4},a_{0})$

Algorithm 2 describes a quantum circuit for Inversion of $F_{2^{8}}/\left(x^{8}+x^{4}+x^{3}+x+1\right)$. The notation CNOT8 means the operation of CNOT gates for 8-qubit arrays. KaratsubaMulL3 is a Level-3 version of the proposed multiplication. Note that the last KaratsubaMulL3 of Algorithm 2 (i.e., line 13) does not clean the ancill qubits. In summary, Inversion of $F_{2^{8}}/\left(x^{8}+x^{4}+x^{3}+x+1\right)$ is implemented by performing KaratusbaMulL3 4 times and Squaring 11 times.
**Algorithm 2** Quantum circuit for Inversion of $F_{2^{8}}/\left(x^{8}+x^{4}+x^{3}+x+1\right)$.**Input:** *a***Output:** $a^{-1}$ ($a^{254}$)//Copy *x* to a1 using 8 CNOT gates1:a← 8-qubit allocation2:CNOT8(a,a1)

//($a\cdot a^{2}$) · $a^{64}$3:a1←Squaring(a1)4:a2←KaratsubaMulL3(a,a1)5:**for** i=0 to 4 **do**6:  a1←Squaring(a1)7:a3←KaratsubaMulL3(a2,a1)

//($a\cdot a^{2}$) · ${\left(a\cdot a^{2}\right)}^{4}$ · $a^{64}$8:**for** i=0 to 1 **do**9:  a2←Squaring(a2)10:a4←KaratsubaMulL3(a3,a2)

//($a\cdot a^{2}$) · ${\left(a\cdot a^{2}\right)}^{4}$ · ${\left(a\cdot a^{2}\right)}^{16}$ · $a^{64}$11:**for** i=0 to 1 **do**12:  a2←Squaring(a2)13:a5←KaratsubaMulL3(a4,a2) //Omit cleaning of ancill qubits

//$\left((a\cdot a^{2}\right)$ · ${\left(a\cdot a^{2}\right)}^{4}$ · ${\left(a\cdot a^{2}\right)}^{16}$ · $a^{64}$${)}^{2}$14:a5←Squaring(a5)15:**return** a5

Table 6 reports the quantum resources needed for the quantum inversion that has been proposed. Compared to stand-alone multiplication (see Table 5), the number of qubits does not increase as expected, even though four multiplications are performed. Since the Toffoli depth of each of the multiplications is one, the Toffoli depth of the inversion is only four.

## 5. Performance

In this section, we summarize the previous research on quantum multiplication and assess the effectiveness of the proposed implementation method.

In a paper by Banegas et al. ([19]), quantum binary field multiplication was introduced as one of the techniques for solving discrete logarithm problems for binary elliptic curves. In this case, quantum multiplication of $F_{2^{n}}$ was implemented using $n^{2}$ Toffoli gates, following the Schoolbook method. To optimize the number of qubits, the authors prioritized the upper products $c^{n}$, $c^{n+1}$, …, $c^{2n-2}$ (reduction part) during the calculation of h=f·g. The result of modular reduction of the upper products is then stored in an *n*-qubit register representing *h*. This method allows for the implementation of quantum multiplication using 3n qubits for *f*, *g*, and *h*. This method employs a minimal number of qubits, but relies on a maximum number of Toffoli gates ($n^{2}$) and has a high Toffoli depth due to its reliance on general Schoolbook multiplication. Table 7 reports the quantum resources needed for the multiplication of $F_{2^{8}}/\left(x^{8}+x^{4}+x^{3}+x+1\right)$ using the Schoolbook multiplication method described in [19].

Kepley et al. in [20] introduced a method for quantum multiplication using the Karatsuba algorithm, which is known to classically reduce the multiplication complexity. The Karatsuba algorithm, which uses the divide-and-conquer method to reduce the number of multiplication operations, was applied to quantum multiplication in [20]. This resulted in a method that uses fewer Toffoli gates for quantum multiplication. Table 7 reports the quantum resources needed for the multiplication of $F_{2^{8}}/\left(x^{8}+x^{4}+x^{3}+x+1\right)$ using the Karatsuba multiplication method described in [20].

Van Hoof in [18] introduced another method for quantum multiplication that uses the Karatsuba algorithm. The method described in [18] used fewer qubits compared to the one presented in [20]. In [20], extra qubits are needed to store the intermediate results of the Karatsuba algorithm. However, the work in [18] employs the LUP decomposition to avoid the need for additional qubits, resulting in a quantum multiplication method that uses the same qubit count as the Schoolbook method (i.e., 3n qubits). In the work of Van Hoof in [18], the number of gates and qubits used in their implementation of the Karatsuba algorithm for quantum multiplication is reported, but the full depth is not given (only reported at the NCT level: NOT, CNOT, Toffoli). The works of [20] and [18] both utilize the Karatsuba algorithm for quantum multiplication, but the latter uses fewer qubits. However, it is assumed that the full depth of the implementation in [18] is higher than that in [20]. This is due to the use of gates in a reduced space hindering parallelism and increasing the depth. According to the estimates in the reference [18], the depth of the field $F_{2^{8}}/\left(x^{8}+x^{4}+x^{3}+x+1\right)$ is 139 at the NCT level, and this depth becomes even greater when the full depth is considered. In Banegas et al.’s work [17], a quantum circuit (Shor’s algorithm) for the binary ECC is constructed using Van Hoof’s quantum multiplication technique.

A comparison of the quantum resources needed for the multiplication of the field $F_{2^{8}}/\left(x^{8}+x^{4}+x^{3}+x+1\right)$ can be found in Table 7. In [18], no exact full depth is given, but it is noted that the NCT depth reported in [18] is greater than the full depth reported in [20].

Recently, we have come across a new approach for space-efficient quantum binary field multiplication [30]. The authors of [30] show that the Toffoli gate count can be further reduced while maintaining the qubit count to a minimum (but the circuit depth is high). The results from [30] are not included in our comparison (i.e., Table 7) because the fields considered and the resource metrics used are different.

Our quantum multiplication method has been optimized to have a Toffoli depth of one for any field size. In quantum multiplication, the Toffoli depth significantly affects the full depth count. Therefore, thanks to the optimized Toffoli depth, our work attains the lowest full depth.

Even though quantum computers in the NISQ era are not necessarily small, they still need quantum error correction. When it comes to error correction, the Toffoli depth metric is likely to be the most critical. However, it is important to consider the trade-off among qubits and depth because the qubit count is still a significant factor. Our quantum multiplication technique requires more qubits on average, but it offers the best compromise between Toffoli depth and the qubit count. This metric (TD·M), where TD is Toffoli depth and *M* is the qubit count, represents the balance for quantum circuits and is used in [3]. Even on another metric representing the trade-off performance, FD·M (FD is full depth), our quantum multiplication achieves the highest performance.

As mentioned in Section 3.3, the increased use of qubits can be compensated for if the multiplication is not a stand-alone multiplication. We have confirmed the effectiveness of the proposed multiplication in quantum inversion based on the Itoh–Tsujii algorithm (see Section 4). Not limited to just inversion, the proposed multiplication can be utilized in a variety of cases where multiplication is used internally in cryptographic operations.

## 6. Conclusions

This paper presents an optimized quantum binary field multiplication technique, which is a key component for quantum cryptanalysis of ECC. Further, this technique can be used for the quantum cryptanalysis of ciphers requiring the multiplication of binary fields.

The aim of our work is the optimization of quantum multiplication to achieve a Toffoli depth of one for any field size. In addition, we present a reverse operation to offset the overhead of ancilla qubits, an optimization with *T*-depth one, and an efficient implementation of modular reduction.

For performance evaluation, our work is compared to previous implementations on various metrics. Furthermore, our work achieves the lowest full depth and provides the best trade-off performance.

Future work is to find another optimization for quantum cryptanalysis building blocks of ECC. As the post-quantum era approaches, the cryptographic community is interested in ways to optimize quantum cryptanalysis. We will note the direction of optimization that should be pursued in quantum implementations.

## Figures and Tables

**Table 1:** Quantum resources required for multiplication at each Karatsuba level.

Field Size $2^{n}$	#CNOT	#Toffoli	Toffoli Depth	#Qubit	Full Depth
Schoolbook	·	$n^{2}$	3n−2	4n−1	8·(3n−2)
Karatsuba Level-1	5n−4	$3\cdot {\left(n/2\right)}^{2}$	3n/2−2	3·(2n−1)	8·(3n/2−2)+5
Karatsuba Level-2	(5n−4)+3·(5n/2−4)	$3^{2}\cdot {\left(n/2^{2}\right)}^{2}$	$3n/2^{2}-2$	$3^{2}\cdot \left(n-1\right)$	$8\cdot (3n/2^{2}-2)+10$
Karatsuba Level-3	(5n−4)+3·(5n/2−4) +9·(5n/4−4)	$3^{3}\cdot {\left(n/2^{3}\right)}^{2}$	$3n/2^{3}-2$	$3^{3}\cdot \left(n/2-1\right)$	$8\cdot (3n/2^{3}-2)+15$

**Table 2:** Quantum resources needed for multiplication with a Toffoli depth of one.

Field Size $2^{n}$	Karatsuba Level	#CNOT	#1qCliff	#*T*	*T*-Depth ^※^	#Qubit	Full Depth
n=4	2	88	18	63	4	27	17
n=8	3	300	54	189	4	81	23
n=16	4	976	162	567	4	243	28
n=127	7	29,298	4370	15,295	4	6555	48
n=163	8	56,642	8774	30,709	4	13,161	52
n=233	8	84,890	12,646	44,261	4	18,969	53
n=283	9	134,370	20,546	71,911	4	30,819	56
n=571	10	410,410	62,342	218,197	4	93,513	61

^※^: Toffoli depth one has a *T*-depth of four.

**Table 3:** Resources required for quantum multiplication at *T*-depth one using AND gates.

Field Size $2^{n}$	Karatsuba Level	#CNOT	#1qCliff	#*T*	*T*-Depth	#Qubit	Full Depth
n=4	2	106	27	36	1	36	16
n=8	3	354	81	108	1	108	22
n=16	4	1138	243	324	1	324	27
n=127	7	33,668	6555	8740	1	8740	47
n=163	8	65,416	13,161	17,548	1	17,548	51
n=233	8	97,536	18,969	25,292	1	25,292	52
n=283	9	154,916	30,819	41,092	1	41,092	55
n=571	10	472,752	93,513	124,684	1	124,684	60

**Table 4:** Results of modular reduction of $F_{2^{8}}/\left(x^{8}+x^{4}+x^{3}+x+1\right)$.

$x^{n}$	Coefficient
n=0	*c*_0_ + *c*_8_ + *c*_12_ + *c*_13_
n=1	*c*_1_ + *c*_8_ + *c*_9_ + *c*_12_ + *c*_14_
n=2	*c*_2_ + *c*_9_ + *c*_10_ + *c*_13_
n=3	*c*_3_ + *c*_8_ + *c*_10_ + *c*_11_ + *c*_12_ + *c*_12_ + *c*_13_ + *c*_14_
n=4	*c*_4_ + *c*_8_ + *c*_9_ + *c*_11_ + *c*_14_
n=5	*c*_5_ + *c*_9_ + *c*_10_ + *c*_12_
n=6	*c*_6_ + *c*_10_ + *c*_11_ + *c*_13_
n=7	*c*_7_ + *c*_11_ + *c*_12_ + *c*_14_

**Table 5:** Resources required for quantum multiplication of $F_{2^{8}}/\left(x^{8}+x^{4}+x^{3}+x+1\right)$.

Field Size $2^{n}$	Karatsuba Level	#CNOT	#1qCliff	#*T*	*T*-Depth	#Qubit	Full Depth
n=8 ^🞲^	3	323	54	189	4	81	32
n=8 ^♢^	3	377	81	108	1	108	31

^🞲^: Using the Toffoli gate decomposition in [31]. ^♢^: Using AND gate.

**Table 6:** Quantum resources needed for inversion of $F_{2^{8}}/\left(x^{8}+x^{4}+x^{3}+x+1\right)$.

Field Size $2^{n}$	Karatsuba Level	#CNOT	#1qCliff	#*T*	*T*-Depth	#Qubit	Full Depth
n=8 ^🞲^	3	1720	216	756	16	162	182
n=8 ^♢^	3	1936	324	432	4	189	174

^🞲^: Using the Toffoli gate decomposition in [31]. ^♢^: Using AND gate.

**Table 7:** Evaluation of quantum resources needed for multiplication of $F_{2^{8}}/\left(x^{8}+x^{4}+x^{3}+x+1\right)$.

Field Size $2^{n}$	Source	#CNOT	#1qCliff	#*T*	Toffoli Depth	#Qubit	Full Depth	TD·M	FD·M
n=8	This work (Section 3.5)	323	54	189	1	81	32	81	2592
	[19]	405	30	448	28	24	216	672	5184
	[20]	270	54	189	8	43	88	344	3784
	[18]	382	54	189	N/A	24	N/A	N/A	N/A

*TD* = Toffoli depth, *M* = number of qubits, *FD* = full depth, N/A = not reported.
